# Ischemic Stroke in Pontine and Corona Radiata: Location Specific Impairment of Neural Network Investigated With Resting State fMRI

**DOI:** 10.3389/fneur.2019.00575

**Published:** 2019-05-31

**Authors:** Chunxiang Jiang, Li Yi, Siqi Cai, Lijuan Zhang

**Affiliations:** ^1^Paul C. Lauterbur Research Center for Biomedical Imaging, Shenzhen Institutes of Advanced Technology, Chinese Academy of Sciences, Shenzhen, China; ^2^Shenzhen College of Advanced Technology, University of Chinese Academy of Sciences, Shenzhen, China; ^3^Department of Neurology Peking University Shenzhen Hospital, Shenzhen, China

**Keywords:** stroke, resting-state fMRI, pontine, corona radiata, regional homogeneity, degree centrality

## Abstract

**Objective:** This study aims to investigate location-specific functional remodeling following ischemic stroke in pons and corona radiata.

**Methods:** This study was approved by the local Institutional Review Board. Written consent was obtained from each of the participants prior to the MRI examination. Thirty six subjects with first ever acute ischemic stroke in pons (PS, *n* = 15, aged 62.8 ± 11.01 years) or corona radiata (CRS, *n* = 21, aged 59.33 ± 13.84 years) as well as 30 age and sex matched healthy controls (HC, *n* = 30, aged 60 ± 6.43 years) were examined with resting state functional magnetic resonance imaging (rs-fMRI). Regional homogeneity (ReHo) and degree centrality (DC) were calculated using a voxel-based approach. Intergroup differences in ReHo and DC were explored using a permutation test with a threshold-free cluster enhancement (PT TFCE, number of permutations = 1,000, family-wise error rate (FWER) < 0.05).

**Results:** ReHo and DC alterations were identified in distributed anatomies for both PS and CRS groups. DC mainly increased in the bilateral anterior and posterior cingulate cortex, the inferior frontal-orbital gyrus, and decreased in the bilateral cuneus, calcarine, and the precuneus, while ReHo mainly decreased in the precentral and the postcentral gyri, inferior parietal lobules, precuneus, posterior cingulate cortex, and the superior occipital gyrus. PS and CRS groups were not significantly different in ReHo or DC (FWER > 0.05).

**Conclusions:** Focal ischemic stroke in pons or corona radiata leads to extensive alterations in the functional network centrality. IS-induced network remodeling is more anatomy-specific than pathway-specific, which may underpin the clinicotopographical profiles during the disease dynamic. Approaches targeting neural pathway and functional connectivity may shed light on a better characterization and management innovation of ischemic stroke.

## Introduction

Despite progress in the disease management and new drug development, the mortality of ischemic stroke (IS) remains high. The overall disability rate is up to 75% in survivors (1). In addition to the disruption to the vascular supply, cerebral ischemia induces extensive structural and functional damages with complex pathophysiological mechanisms (2). Studies with an unspecified IS location may be confounded by the heterogeneity of the functional specification (3–5). Lesion inference analysis revealed that the functional outcome of IS corresponds to brain areas and vascular territory with causal attributions to the behavioral and cognitive consequences (6, 7). The functional impairment associated with IS in different anatomies varies among individuals (8, 9), but may induce a similar clinical profile. The functional reorganization of the brain with IS and the overall outcome may be associated with the lesion location across subjects with IS. The underlying mechanism is not fully understood.

Pontine and corona radiata are located in the same projections between the cortex and brain stem. They are the most common sites of IS that involve the subcortical motor pathway. It is well-known that focal IS leads to extensive structural and functional remodeling (10, 11). However, it remains unclear whether pontine stroke (PS) and corona radiata stroke (CRS) differ in the patterns of the functional remodeling. Decoding this information would be helpful to disclose the neural mechanisms of IS toward designing individualized protocol for disease management and optimizing the neuroimaging strategy for the evaluation of the treatment response and the prediction of the functional outcomes.

Blood oxygen-level-dependent functional magnetic resonance imaging (BOLD fMRI) has become a popular technique for the investigation of brain function in healthy (12–14) and diseased human brains (15, 16). However, perfusion and oxygenation remodeling in the perilesional area and regions distant to the IS lesion may substantially confound the neurovascular coupling, and neural activity changes after IS may not be well-represented based on the canonical hemodynamic response function. Alternatively, network-based approaches derived from the resting-state fMRI (rs-fMRI) are promising in the estimation of the IS- induced functional variations due to its independence of task performance (17). Regional homogeneity (ReHo) is an index derived from rs-fMRI assessing the synchrony of neural activity in a given area (18) and degree centrality (DC) weighting the importance of brain regions within the entire brain network (19). An investigation of the post-stroke neural reorganization from the perspective of elements and connections may provide insight complementary to the established physiopathology of IS. In this study, we aim to investigate the location-specific functional alteration of the neural network in brains with PS and those with CRS relative to the healthy controls using ReHo and DC approaches, based on resting-state functional magnetic resonance imaging (rs-fMRI).

## Materials and Methods

### Participants

This study was approved by the local Institutional Review Board. Written consent was obtained from each of the participants prior to the MRI examination. Subjects with clinically confirmed IS from December 2012 to June 2013 were included with the following criteria: (a) right-handed; (b) first ever single lesion in PS or CRS; (c) MRI data acquired within 2 weeks from the onset of neurological deficit; (d) no other abnormalities in brain parenchyma on MR images; (e) no history of neurological or psychiatric illness, resulting in 36 subjects (15 with PS, 21 with CRS) who qualified for the study. The National Institutes of Health Stroke Scale (NIHSS) was used to quantify the functional impairment caused by IS. In addition, 30 age and sex matched healthy subjects were recruited as controls (HC).

### Data Acquisition

Structural and functional imaging data were acquired with a 1.5T imaging system (Siemens, Erlangen, Germany) with a 12 channel phased array head coil. Resting state fMRI images were acquired using an echo-planar imaging (EPI) sequence with typical parameters of TR/TE 3,000/30 ms, flip angle 90°, field of view 210 mm, matrix 128 × 128, slice thickness 3 mm, bandwidth 1,395 Hz/pixel, 60 volumes, resulting in a scan time of 3 min. Subjects were instructed to relax their minds, with their eyes closed remaining motionless as much as possible during the data acquisition. In addition, high resolution T1-weighted images were obtained using 3D MPRAGE with TR/TE/TI 1,900/2.53/900 ms, flip angle 9°, FOV 250 mm, in-plane resolution 1.0 × 1.0 × 1.0 mm. T2-weighted images were obtained with parameters of TR/TE 4,000/89 ms, flip angle 150°, FOV 250 mm, in-plane resolution 0.3 × 0.3 mm × 5.0 mm. Diffusion weighted imaging was acquired with a single-shot spin echo EPI sequence and the typical parameters were TR/TE 4,100/100 ms, FOV 250 mm, image matrix 128 × 128, slice thickness 3 mm, and a non-zero b factor of 1,000 s/mm^2^ applied in 20 gradient directions.

### Data Preprocessing

Data preprocessing was conducted using DPARSF (Data Processing Assistant for Resting-State fMRI, http://www.restfmri.net) which is based on Statistical Parametric Mapping (SPM8, http://www.fil.ion.ucl.ac.uk/spm) and the Resting-State fMRI Data Analysis Toolkit (http://www.restfmri.net). To reduce the impact of lesions on segmentation and normalization, stroke lesions were masked out during the segmentation and normalization procedures. Preprocessing procedures included: 1) removal of the first 10 volumes; 2) slice dependent time shifts; 3) motion correction; 4) T1-weighted images co-registered to the mean functional image and segmented into gray matter (GM), white matter (WM) and cerebrospinal fluid (CSF) tissue maps; 5) functional images normalized to the MNI space using the transformation parameters and resampled to 3 × 3 × 3 mm^3^; 6) spatial smoothing with a 4 mm full-width half-maximum Gaussian filter; 7) band-pass temporal filtering (0.01–0.08 Hz); 8) removing linear trends; 9) regression of nuisance variables, including Friston 24-parameter model (i.e., six head motion parameters, six head motion parameters one time point before, and the 12 corresponding squared items) (20), as well as five principal components extracted from each subject's WM and CSF mask, using a component based noise correction method (CompCor) (21).

To further explore the residual effect of motion on fMRI measures, volume-level mean framewise displacement (FD) was computed (22). Three subjects (one in each group) with a mean FD greater than mean + 2SD were excluded, with a threshold of 0.1426 mm for the HC group, 0.2262 mm for the PS group, and 0.1536 mm for the CRS group, respectively. The average mean FD in the HC group was 0.0923 mm (*SD* = 0.0499 mm, range = 0.0367–0.1238 mm), 0.0811 mm (*SD* = 0.0294 mm, range = 0.0394–0.1537 mm) in the PS group, and 0.0923687 mm (*SD* = 0.0271 mm, range = 0.0350–0.1203 mm) in the CRS group. Differences of mean FD among the three groups were tested using one-way ANOVA which showed no significant difference (*F* = 2.075, *p* = 0.135).

### Parameter Calculation

ReHo was defined by Kendall's coefficient of concordance (KCC) which was used to measure the similarity between the time series of a given voxel and its nearest neighbors (27 voxels in this study) (18, 23). In order to reduce the effect of individual variability, ReHo was divided by the global mean value for each subject. Degree centrality (DC) calculates the correlation between the BOLD time series of each voxel and all the other voxels in the gray matter mask. A Fisher transformation was applied to improve the normality of the maps mentioned above. The threshold for DC was set at 0.25 to eliminate weak correlations (24–26). DC maps with alternative thresholds can be found in the Supplementary Materials.

### Lesion Distribution

**Figure 1:**
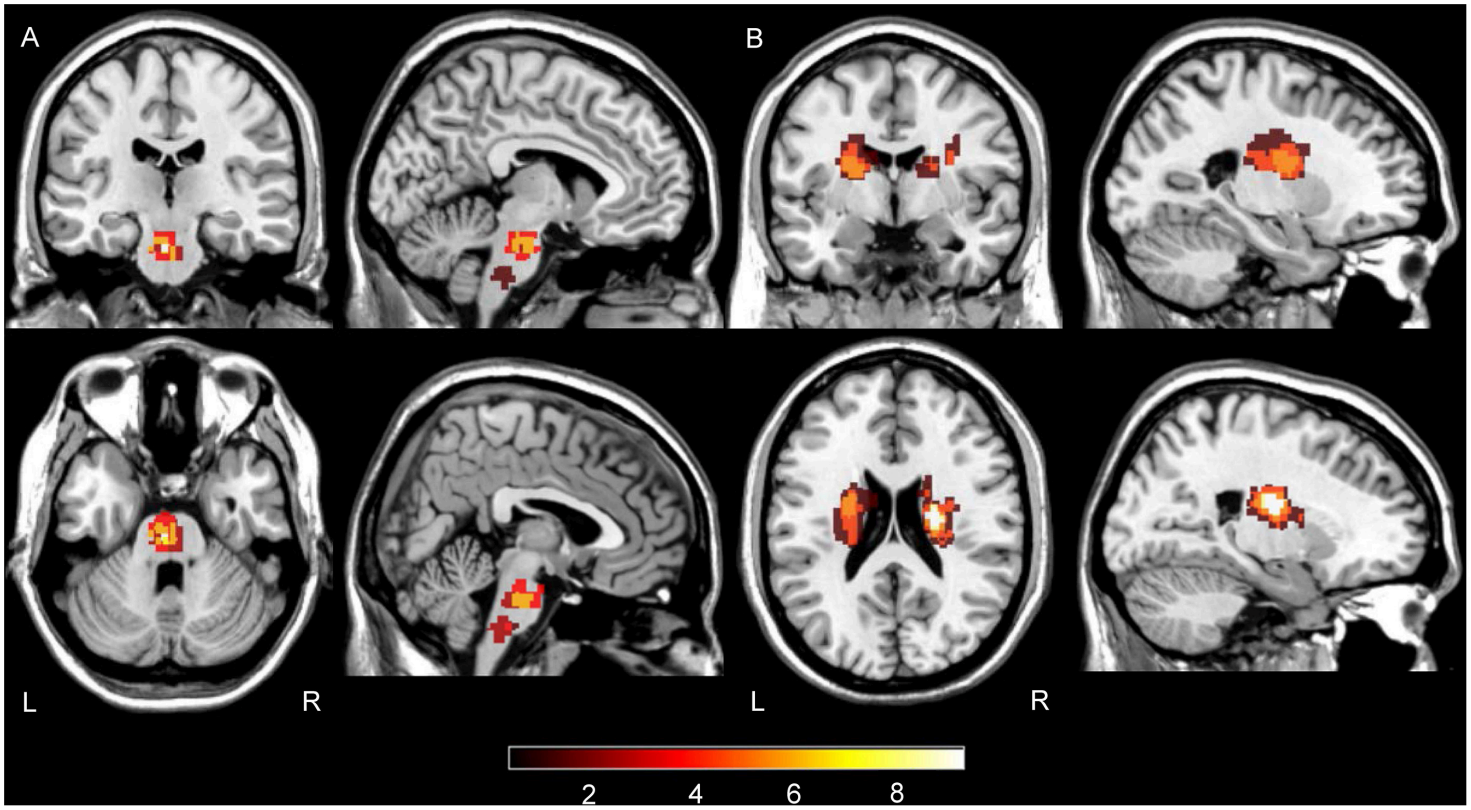
Summation maps of lesion distribution. **(A)** Lesion distributions in PS patients. **(B)** Lesion distributions in CRS patients. Color bar denotes the summation distribution of lesions. L, left hemisphere; R, right hemisphere.

Stroke lesions were manually traced, by L Zhang who has 7 years of experience as a Neuroradiologist and 17 years as an investigator of brain research using MRIcron software (www.nitrc.org/projects/mricron), based on the diffusion weighted images in individual spaces and extracted as lesion masks. Then the individual diffusion images were co-registered to the standard MNI space and the derived transformation parameters were used to transform lesion masks to the MNI space. The transformed lesion masks were overlaid to obtain the lesions summation map and represented on a standard T1-weighted image template (Figure 1).

### Statistical Analysis

One-way ANCOVA (analysis of covariance) was used to compare the differences of ReHo and DC among the PS, CRS and HC groups with FD as covariates. Given the relatively small sample size per group, a permutation test with a threshold free cluster enhancement (PT TFCE, number of permutations = 1,000, family-wise error rate (FWER) < 0.05) was adopted to extract the brain regions with a significant difference among the three groups which was used as a mask in the *post-hoc* two sample *t*-test between each group pair. According to a recent study, PT TFCE is a strict multiple comparison correction strategy, which reaches relatively lower FWER and higher reliability/replicability (27, 28). Also, the PT TFCE (number of permutations = 1,000, FWER < 0.05, two tailed) method was used in the *post-hoc* two-sample *t*-test, with FD as a covariate. Pearson's correlation was used to determine the degree of correlation between the NIHSS scores and ReHo, as well as DC values within PS and CRS groups, respectively, and corrected with PT TFCE (number of permutations = 1,000, FWER < 0.05, two tailed). The Chi-square test and one-way ANOVA were used to compare the differences of sex and age among the three groups (SPSS, version 19).

## Results

The demographics of the subjects in this study are summarized in Table 1. The time from the on-set of neurological deficits to MR imaging (T) and FD were not statistically different between the CRS and PS groups (*p* > 0.05). NIHSS score was not correlated with ReHo or DC after being PT TFCE corrected (*p* > 0.05).

**Table 1:** Summary of demographics of all subjects.

**Characteristic**	**PS**	**CRS**	**HC**	***P*-value**
n	15	21	30	–
Age (y)^*^	62.8 ± 11.01	59.33 ± 13.84	60 ± 6.43	0.51 (*F* = 0.673)
Sex (M/F)	10/5	16/5	17/13	0.351 (χ^2^ = 2.094)
Time from onset to imaging (T) (h)^*^	40.5 ± 25.5	74.6 ± 60.6	–	0.102
NIHSS^*^	3.27 ± 3.34	3.57 ± 2.94	–	0.77

CRS, corona radiata stroke group; HC, healthy control group; NIHSS, National Institutes of Health Stroke Scale; PS, pontine stroke group.

* *Data are means ± standard deviation*.

### Alterations of DC in PS and CRS Groups Relative to HC

**Figure 2:**
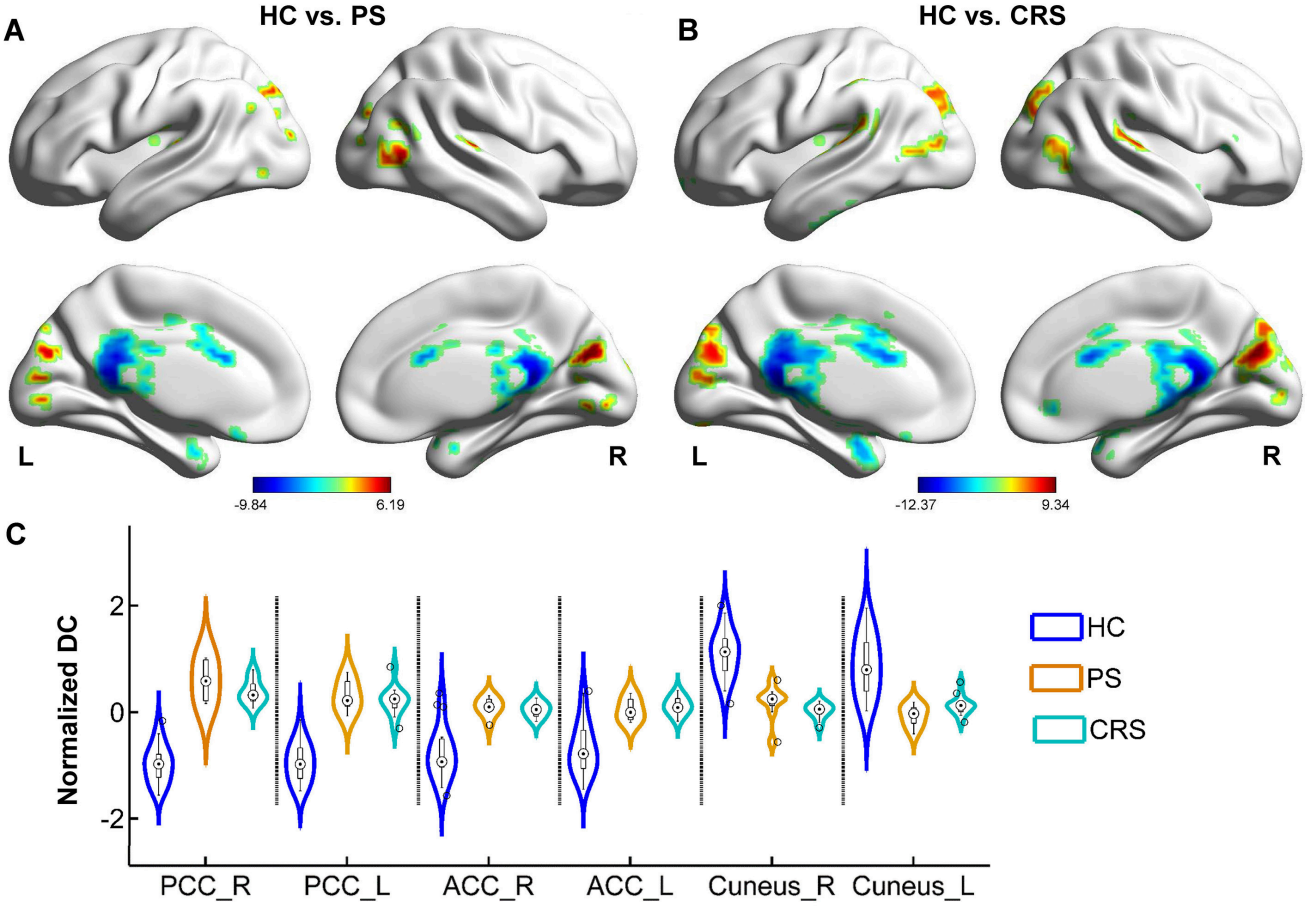
DC differences between PS, CRS and HC groups. Brain regions with significant differences between HC and PS group **(A)**, HC and CRS group **(B)**. Color bar denotes t value (FWER < 0.05, TFCE corrected). **(C)** The distribution of normalized DC in statistically significant clusters in each group. PCC, posterior cingulate cortex; ACC, anterior cingulate cortex.

Relative to that of the HC group, DC in both PS and CRS groups exhibited a significant increase mainly in the bilateral posterior cingulate cortex (PCC), anterior cingulate cortex (ACC), temporal pole, and the inferior frontal-orbital gyrus, and a decrease in the bilateral cuneus, precuneus (PCU), superior, and the middle temporal gyri (Figure 2).

### Alterations of ReHo in PS and CRS Groups Relative to HC

**Figure 3:**
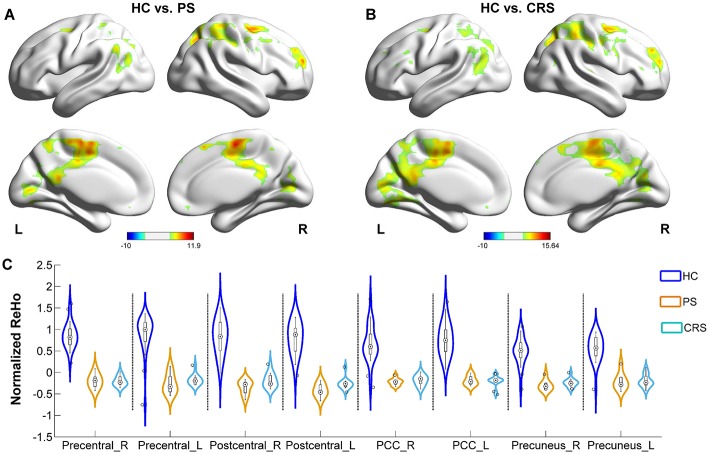
ReHo differences between PS, CRS and HC groups. **(A)** Brain regions with significant differences between PS and HC group. **(B)** Brain regions with significant differences between CRS and HCs group. Color bar denotes *t*-value (FWER < 0.05, TFCE corrected). **(C)** ReHo distribution in bilateral precentral gyrus, postcentral gyrus, posterior cingulate cortex, and precuneus.

Relative to the HC group, PS and CRS showed a decreased ReHo in the sensorimotor cortex network components, including the bilateral precentral and the postcentral gyri, the default mode network components including the inferior parietal lobules, PCU and PCC. In addition, the middle occipital gyrus, middle temporal gyrus, superior and the middle frontal gyri also showed a decreased ReHo in PS and CRS groups compared to HC (Figure 3).

Notably, there was no difference found in DC and ReHo maps between the PS and CRS groups after PT TFCE analysis with FWER < 0.05.

## Discussion

The functional response of the brain to the ischemic attack in various lesion locations is critical to consider when designing treatment and rehabilitative strategies for IS. This study examined the differences in the intrinsic neural activity and connectivity pattern of the whole-brain functional networks in patients with pontine and corona radiata IS with voxel-wised ReHo and DC approaches. Pons coordinate sensory relay between the cerebrum and cerebellum, while corona radiata carries somatotopically arranged motor fibers connecting the cortex and brain stem. Damage to the pathway routing corona radiata and pons disconnect the associated functional circuitry and leads to variable alterations to the large-scale brain networks. Both the PS and CRS groups showed extensive connectivity alterations with anatomy specifications, supporting that ischemic stroke is a disease of network disintegration in addition to the focal vascular failure.

### Altered Functional Connectivity in PS and CRS Groups

As a deputy parameter weighting the element importance of the brain network, DC was found altered for both the PS and CRS groups in distributed brain areas including the bilateral PCC, ACC, left inferior frontal-orbital gyrus, bilateral temporal pole, superior and middle temporal gyri, bilateral cuneus and the PCU gyri. This suggests a similar pattern of brain network responses to IS in the pons and corona radiata. Particularly, the increased DC in ACC, PCC in both groups may indicate substantial alterations in the hierarchical organization of the brain functional networks with an increased importance of the default-mode network (DMN), as anatomies with higher DC contribute more to the overall network efficiency. This finding was in agreement with a previous study that the anterior DMN showed increased activity in IS (29). DMN components are also recognized as brain hubs with higher energy metabolic demands and topological values (30–32). The increased weighted importance of DMN may suggest a compensatory process for the information flow coordination and network integrity maintenance, counteracting the IS-induced functional loss that may dissociate from the location specificity of infarct topology (33, 34).

The corona radiata consists of massive fiber bundles connecting with the ACC and inferior frontal-orbital gyrus. These structures are among the pivotal hubs for the neural circuitry in charge of voluntary emotional expression and cognition processing. Damage to the corona radiata disconnects the functional circuitry between the frontal cortex and brain stem, disturbing voluntary emotional expression. Psychological and psychiatric consequences were commonly observed in the PS and CRS subjects (35–37). ACC was reported to show increased DC in subjects with depression (38). The increased DC in the ACC and inferior frontal gyrus in the CRS group of this study, may be suggestive of preclinical post-stroke depression of the CRS subjects.

About one third of the subjects with IS suffer from post-stroke visual impairment, which is less evident than the impairment of motor and speech functions (39, 40). DC was reported to decrease in the cuneus in subjects with damage in the visual pathway (41). Since not all subjects of this study reported vision impairment at the time of MRI data acquisition, the decreased DC in the cuneus in this study may represent an adaptation mechanism counteracting the extensive network alterations, in addition to the possible late onset impairment of visual processing. A follow-up investigation would be helpful to confirm the significance of altered connectivity to the cuneus in predicting the visual consequence after IS.

### Altered ReHo in PS and CRS Groups

ReHo evaluates the synchrony of neural activity in a given area. Voxels with higher ReHo are more active in synchronizing with a large number of neighboring neurons. A previous study showed that decreased ReHo in the PCC/PCU region was related to the impairment in consciousness in subjects with acute IS (42). Decreased ReHo in DMN components including the PCC/PCU and the inferior parietal lobules in this study may suggest a reduced neuronal recruitment in the coordination of conscious awareness, sensory interpretation and attention salience. As a central node in the DMN, PCC has structural connectivity with widespread brain regions and communicates with various brain networks (43), the opposite alterations in DC and ReHo in PCC may indicate the additional recruitment of neural circuits in response to the extended network disruption during the post-stroke functional remodeling. In addition, ReHo was found to decrease in the middle temporal and superior occipital gyri in subjects with IS below the thalamus (44), which is likely attributed to the nodal impairment of the cortico-thalamo-cortico loop. The ReHo decrease in superior occipital, middle temporal gyri and superior/middle frontal gyrus in this study may partially underlie the compromised visual auditory kinetics and memory processing, which was commonly observed in the PS and CRS subjects. Furthermore, both PS and CRS groups showed ReHo decrease in the sensorimotor cortices in this study. This may substrate the disruption of sensorimotor coordination that is not fully interpretable by the lesion itself for the IS subjects (45). A previous study showed increased ReHo in the non-somatosensory areas of the ipsi- or contralesional hemisphere during motor recovery in the chronic stage of IS (46), implying that ReHo in the non-affected brain area is indicative of the disease dynamic of IS.

### Speculations on the Lack of Difference in Neural Activity Between PS and CRS Groups

Pons and corona radiata are located in the same projection between the cortex and the brain stem, but they involve the nodal elements of the sensory-motor circuit at a different level. It is reasonable to speculate that the PS and CRS groups showed different patterns of neural activity variation indexed by DC and ReHo, as shown in this study. However, the numerical comparison did not show a significant PS-CRS difference in the ReHo and DC quantification. Multiple factors may contribute to this finding. First, although the pons and corona radiata belong to a pathway with the same structural connection between the cortex and brain stem, the IS-induced variations in the network centrality indexed by ReHo and DC may be more anatomy-specific than pathway specific. In addition, ReHo is frequency dependent for the characterization of the nodal importance in the local functional interaction (47). Further studies of ReHo at different frequency bands would enable a better investigation of the location specific alteration of the neural activity after IS. Second, effects of stroke laterality were not considered for the group level analysis. Stroke laterality was reported to bias the disease characterization of IS, although the overall 10-year survival rate does not differ between IS in the left vs. right hemisphere (48). Third, patients' rs-fMRI data was collected within 2 weeks after the disease onset, during which the interaction of the functional impairment and remodeling may not have fully developed at the time of neuroimaging. A longitudinal follow-up study would be helpful in exploring the chronological pattern of local and systemic neural response of IS. Fourth, the effect of post-stroke cortical atrophy may also confound the neural activity analysis, but its confounding effect was believed to be limited in the current highly selected cohort, since remote cortical atrophy was observed in chronic instead of acute IS subjects (49). Last, the scan length was reduced to 3 minutes in this study, which may hinder the reliability of functional connectivity estimates (50) that differentiates the neural activity alterations between the PS and CRS groups, especially in the context of the IS-induced neuro-vascular uncoupling at the global scale. Some patients in this cohort suffered from dystonia with involuntary and repetitive movements, which could fail the scan during a long acquisition time. Van Dijk et al. (51) highlighted that runs shorter than 4 min may reduce the sensitivity. Whitlow et al. (52) examined the stability of FC estimates using a TR of 2 s and showed that as little as 1.5–2 min of rs-fMRI is sufficient to accurately compute FC with the equivalent number of volumes in this study. Shah et al. (53) reported that the reproducibility of the connectivity estimates for rs-fMRI is a function of the square root of imaging time, and a scan length at or <2 min is inadequate to construct meaningful functional connectivity estimates. It is therefore worth exploring the optimization of the rs-fMRI data acquisition for IS subjects in future work.

## Conclusions

Focal IS in the pons or corona radiata leads to extensive alterations in functional network centrality in addition to a focal vascular failure. IS-induced network remodeling is more anatomy-specific than pathway-specific, which may underpin the clinicotopographical profiles during the disease dynamic. Approaches targeting neural pathways and functional connectivity may shed light on better disease characterization and innovation in IS management.

## Data Availability

The datasets generated for this study are available on request to the corresponding author.

## Ethics Statement

This study was carried out in accordance with the recommendations of Shenzhen Institutes of Advanced Technology Review Board. All subjects gave written informed consent in accordance with the Declaration of Helsinki. The protocol was approved by Shenzhen Institutes of Advanced Technology Review Board.

## Author Contributions

CJ and LZ designed this study and wrote the manuscript. CJ, LY, and SC performed the data analysis. All authors approved the final version of the article.

### Conflict of Interest Statement

The authors declare that the research was conducted in the absence of any commercial or financial relationships that could be construed as a potential conflict of interest.
