# Supplementary material for: Ischemic Stroke in Pontine and Corona Radiata: Location Specific Impairment of Neural Network Investigated With Resting State fMRI
Source: Front Neurol. 2019 May 31;10:575. doi: 10.3389/fneur.2019.00575 (PMC6554416; doi:10.3389/fneur.2019.00575)
Supplement: Supplementary file 1 [file Data_Sheet_1.docx]

Supplementary Material

**Ischemic Stroke in Pontine and Corona Radiata: Location Specific Impairment of Neural Network Investigated with Resting State fMRI**

**Chunxiang Jiang ^1, 2^, Li Yi ^3^, Siqi Cai ^1^, Lijuan Zhang^1*^**

^1^Paul C. Lauterbur Research Center for Biomedical Imaging, Shenzhen Institutes of Advanced Technology, Chinese Academy of Sciences, Shenzhen, China

^2^Shenzhen College of Advanced Technology, University of Chinese Academy of Sciences, Shenzhen, China.

^3^Department of Neurology, Peking University Shenzhen Hospital, Shenzhen, China

- **Degree centrality maps at alternative thresholds**

Degree centrality maps were thresholded at r = 0.25, consistent with previous studies (Takeuchi et al., 2015; Gao et al., 2016; Xiao et al., 2016). As a further test on the robustness of the weighted degree centrality results, we calculated degree centrality maps at alternative thresholds (r = 0.20 and r = 0.30). This did not qualitatively affect the results as shown in Fig. S1.


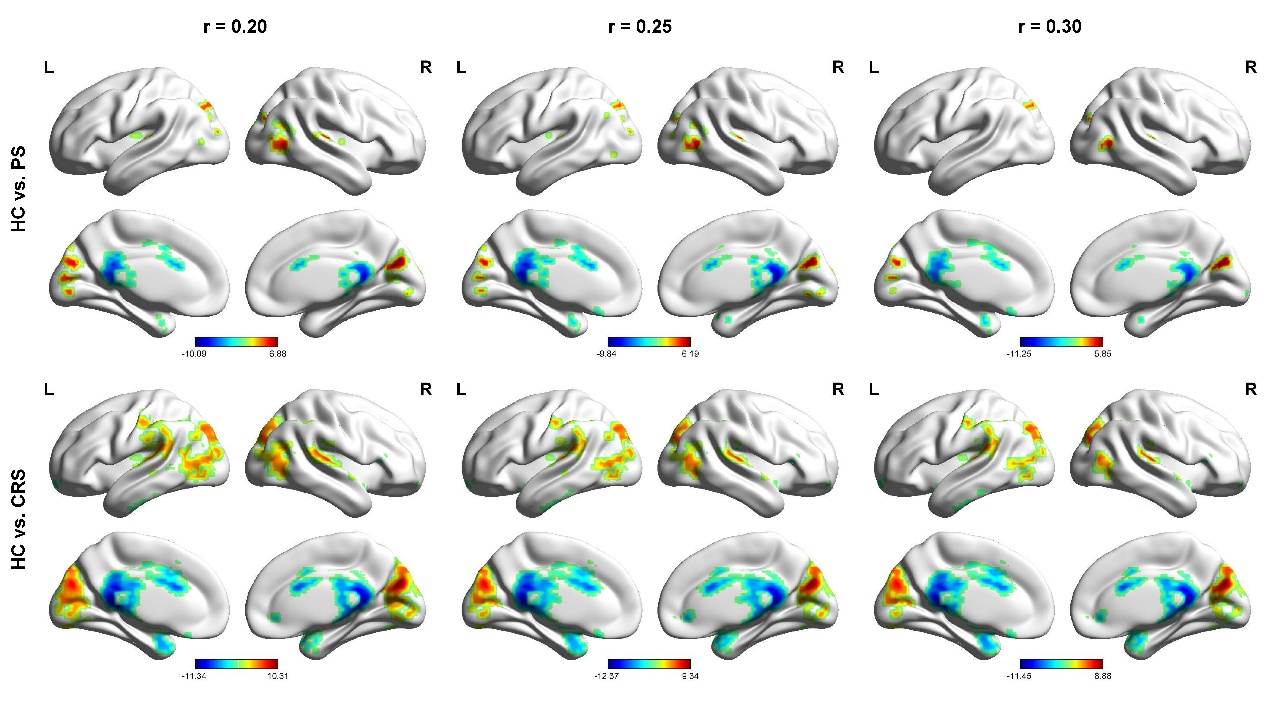


Fig. S1 Group differences of degree centrality between HC and PS groups(first row), HC and CRS groups(second row) are shown at different threshold(left column: r = 0.20; middle column: r = 0.25; right column: r = 0.30).

Table S1. Summary of neuroanatomical regions with significant DC differences between PS/CRS and HC groups.

| Subgroup | Region | Peak MNI  Coordinates  (x, y, z) | voxels | Mean DC value | |
| --- | --- | --- | --- | --- | --- |
|  |  |  |  | Healthy group | Patient group |
| HC  vs.  PS | Cingulum_Ant_R | 6,16,25 | 24 | -0.76857 | 0.0656959 |
|  | Cingulum_Ant_L | -5,16,25 | 30 | -0.701663 | 0.080111 |
|  | Cingulum_Post_R | 6,-42,22 | 57 | -0.821621 | 0.157659 |
|  | Cingulum_Post_L | -3,-42,24 | 85 | -0.830472 | 0.244677 |
|  | Cuneus_R | 7,-80,27 | 47 | 1.04929 | 0.192526 |
|  | Cuneus_L | -3,-81,27 | 26 | 1.02759 | 0.0909412 |
|  | Precuneus_R | 6,-48,14 | 34 | -0.824586 | 0.216462 |
|  | Precuneus_L | -6,-46,14 | 38 | -0.779114 | 0.0431778 |
|  | Temporal_Pole_R | 22,10,-40 | 14 | -0.521244 | 0.10287 |
|  | Temporal_Pole_L | -22,8,-40 | 12 | -0.518734 | 0.142145 |
|  | Temporal_Sup_R | 51,-28,9 | 22 | 0.915569 | 0.0739448 |
|  | Temporal_Sup_L | -48,-26,6 | 13 | 0.994591 | 0.115914 |
|  | Temporal_Mid_R | 51,-66,7 | 20 | 0.887552 | 0.126175 |
|  | Temporal_Mid_L | -51,-64,6 | 13 | 0.879435 | 0.186937 |
|  | Frontal_Inf_orb_L | -40,51,-12 | 10 | -0.359801 | 0.24986 |
| HC  vs.  CRS | Cingulum_Ant_R | 5,15,25 | 33 | -0.76857 | 0.144601 |
|  | Cingulum_Ant_L | -5,15,25 | 50 | -0.701663 | 0.102223 |
|  | Cingulum_Post_R | 5,-40,21 | 69 | -0.747395 | 0.10688 |
|  | Cingulum_Post_L | -3,-43,21 | 83 | -0.865804 | 0.147595 |
|  | Cuneus_R | 8,-78,26 | 113 | 1.13283 | 0.0594404 |
|  | Cuneus_L | -7,-80,26 | 70 | 1.11426 | 0.149036 |
|  | Precuneus_R | 7,-49,14 | 48 | -0.824586 | 0.203617 |
|  | Precuneus_L | -4,-49,14 | 43 | -0.880568 | 0.236486 |
|  | Temporal_Pole_R | 36,10,-28 | 17 | -0.449862 | 0.194483 |
|  | Temporal_Pole_L | -20,8,-32 | 38 | -0.611597 | 0.117096 |
|  | Temporal_Sup_R | 48,-28,11 | 72 | 0.978417 | 0.119515 |
|  | Temporal_Sup_L | -47,-28,11 | 65 | 0.999253 | 0.204822 |
|  | Temporal_Mid_R | 50,-68,9 | 21 | 0.859618 | 0.215585 |
|  | Temporal_Mid_L | -52,-64,6 | 28 | 0.898968 | 0.16483 |
|  | Frontal_Inf_orb_L | -39,51,-13 | 10 | -0.383792 | 0.20267 |

Table S2. Summary of neuroanatomical regions with significant ReHo differences between PS/CRS and HC groups.

| Subgroup | Region | Peak MNI  Coordinates  (x, y, z) | voxels | Normalized Mean ReHo value | |
| --- | --- | --- | --- | --- | --- |
|  |  |  |  | Healthy group | Patient group |
| HC  vs.  PS | Precentral_R | 17,-24,64 | 276 | 0.853242 | -0.182738 |
|  | Precentral_L | -23,-23,62 | 258 | 0.833767 | -0.291229 |
|  | Postcentral_R | 16,-38,64 | 407 | 0.665577 | -0.253149 |
|  | Postcentral_L | -22,-35,63 | 128 | 0.821447 | -0.293822 |
|  | Cingulum_Post_R | 6,-44,22 | 64 | 0.728248 | -0.144827 |
|  | Cingulum_Post_L | -6,-46,22 | 85 | 0.696922 | -0.219658 |
|  | Precuneus_R | 10,-56,28 | 167 | 0.549887 | -0.0912873 |
|  | Precuneus_L | -5,-55,28 | 260 | 0.627191 | -0.0316523 |
|  | Frontal_Sup_R | 7,21,55 | 207 | 0.535787 | -0.189459 |
|  | Frontal_Sup_L | -15,19,50 | 134 | 0.494933 | -0.34430 |
|  | Frontal_Mid_L | -33,19,46 | 179 | 0.338421 | -0.377926 |
|  | Frontal_Mid_R | 39,15,46 | 141 | 0.450032 | -0.27771 |
|  | Parietal_Sup_R | 24,-43,59 | 203 | 0.847369 | -0.318084 |
|  | Parietal_Sup_L | -18, -48,62 | 201 | 0.810859 | -0.297453 |
|  | Parietal_Inf_R | 45,-46,43 | 208 | 0.578494 | -0.201514 |
|  | Parietal_Inf_L | -50,-38,41 | 255 | 0.438852 | -0.287026 |
|  | Angular_L | -52,-56,34 | 190 | 0.447796 | -0.200636 |
|  | Occipital_Sup_R | 25,-71,42 | 92 | 0.576326 | -0.174166 |
|  | Occipital_Sup_L | -22,-68,36 | 76 | 0.552223 | -0.300537 |
| HC  vs.  CRS | Precentral_R | 35,-9,57 | 337 | 0.601735 | -0.284046 |
|  | Precentral_L | -29,-14,53 | 212 | 0.370531 | -0.302409 |
|  | Postcentral_R | 25,-38,60 | 446 | 0.505018 | -0.256183 |
|  | Postcentral_L | -24,-36,60 | 126 | 0.40733 | -0.256414 |
|  | Cingulum_Post_R | 4,-39,32 | 74 | 0.352112 | -0.202359 |
|  | Cingulum_Post_L | -6,-22,40 | 110 | 0.804005 | -0.303538 |
|  | Precuneus_R | 8,-63,42 | 368 | 0.684729 | -0.260887 |
|  | Precuneus_L | -8,-60,35 | 454 | 0.588721 | -0.241571 |
|  | Frontal_Sup_R | 14,18,52 | 309 | 0.514320 | -0.268379 |
|  | Frontal_Sup_L | -15,18,52 | 128 | 0.494933 | -0.34430 |
|  | Frontal_Mid_R | 39,16,43 | 319 | 0.393383 | -0.243675 |
|  | Frontal_Mid_L | -31,24,42 | 188 | 0.489912 | -0.319363 |
|  | Parietal_Sup_R | 29,-48,59 | 211 | 0.512594 | -0.208498 |
|  | Parietal_Sup_L | -28,-50,57 | 229 | 0.463654 | -0.213594 |
|  | Parietal_Inf_R | 45,-42,48 | 209 | 0.544187 | -0.217593 |
|  | Parietal_Inf_L | -33,-43,48 | 304 | 0.363992 | -0.283603 |
|  | Cuneus_R | 10,-75,21 | 114 | 0.601735 | -0.230535 |
|  | Cuneus_L | -6,-80,19 | 136 | 0.370531 | -0.208984 |
|  | Angular_L | -45,-53,37 | 224 | 0.505018 | -0.174325 |
|  | Occipital_Sup_R | 24,-73,37 | 109 | 0.472685 | -0.208651 |
|  | Occipital_Sup_L | -21,-75,35 | 97 | 0.512801 | -0.258243 |
|  | Occipital_Mid_L | -35,-75,16 | 290 | 0.40733 | -0.22431 |
|  | Temporal_Mid_L | -55,-48,3 | 139 | 0.352112 | -0.215605 |
